# A tale of excess: the curious case of the woman with 1447 emergency visits

**DOI:** 10.1192/j.eurpsy.2023.644

**Published:** 2023-07-19

**Authors:** M. Sagué Vilavella, A. Giménez Palomo, A. Àvila-Parcet, T. Fernández Plaza, L. Navarro Cortés, G. Oretega Hernández, M. Pons Cabrera, L. Tardón Senabre, M. Vázquez

**Affiliations:** ^1^Department of Psychiatry and Psychology, Hospital Clínic; ^2^Department of Psychiatry, Hospital de la Santa Creu i Sant Pau; ^3^Department of Psychiatry, Hospital Vall d’Hebrón, Barcelona, Spain

## Abstract

**Introduction:**

Frequent attenders to emergency services are challenging and costly. We report the case of a woman in her mid-twenties who stands out for a total of 1447 emergency visits.

**Objectives:**

Our primary objective was to describe the emergency visits of our patient. Secondary objectives were to assess her use of other healthcare services and to calculate her health expenditure.

**Methods:**

This is a clinical case report. We reviewed the patient’s electronic medical records for sociodemographic and clinical data. We obtained detailed information of psychiatric ED visits (length, most frequent times and days) regarding the second most-visited hospital. We assessed the efficacy of hospitalizations in reducing ED visits with a paired samples t Test, comparing the number of visits 30 days pre- and post-hospitalization. We estimated the health expenditure using the regional public health system prices, including three direct costs: emergency visits, hospitalizations and ambulance transportation. We obtained written informed consent from the patient’s legal guardian.

**Results:**

A 26-year-old woman from Barcelona (Catalonia, Spain), diagnosed with mild intellectual disability, made 1447 emergency visits between 2009 and 2021 (figure 1). 946 visits (65%) took place in psychiatric emergency departments (EDs), whilst 353 (24%) in non-psychiatric EDs and 148 (10%) in urgent primary care. She attended 24 hospitals (ranking number one the closest to the patient’s home, with 387 visits) and seven primary care centers, distributed across 17 cities in Catalonia. Most visits were self-referred, being the main presenting problems anxiety and instrumental suicidal behaviour. Saturday was her favorite day for hospital visits (24,1%), while she seeked care on Tuesdays much less often (4.5%). She made 73.5% of consultations between 1pm and 6pm, with a median length per visit of 2.8 hours (range 0.33-20.9 hours). Regarding other therapeutic approaches, she attended day hospitals, psychiatric rehabilitation programs and family therapy, among others (figure 2), for which she showed low adherence and scarce benefit. She had ten acute hospitalizations, interventions that did not reduce ED visits (t=-0.9835, p=0.36). Health expenditure reached 410.035€.

**Image:**

**Figure:**
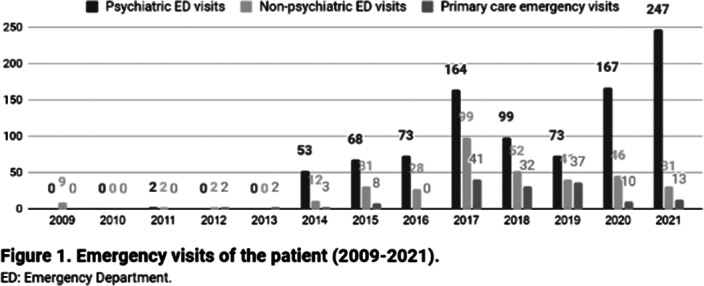

**Image 2:**

**Figure:**
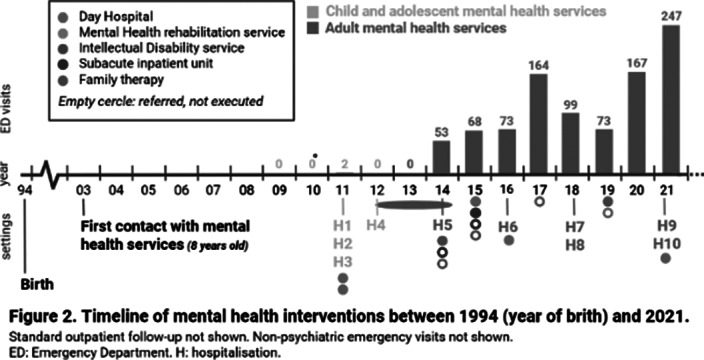

**Conclusions:**

The most common definition of frequent attendance is a patient who has five or more visits per year. Many times, but not always, repeat visits are also inappropriate. The case we report is a grotesque example of both frequent and inappropriate attendance, which has been resistant to all kinds of interventions and has quality-of-care, financial and ethical implications. As of today, it is still a pending case. Maybe it is worth considering residential treatment?

**Disclosure of Interest:**

None Declared

